# Modelling simultaneous detection of electrons and $\gamma$ rays in-beam

**DOI:** 10.1140/epja/s10050-025-01745-9

**Published:** 2025-11-24

**Authors:** D. M. Cox, P. Papadakis, A. D. Briscoe, A. M. Plaza, J. Ojala, J. Pakarinen

**Affiliations:** 1 Oliver Lodge Laboratory, University of Liverpool, Liverpool, L69 7ZE United Kingdom; 2 Department of Physics, University of Jyväskylä, Accelerator Laboratory, P.O. Box 35, FI-40014 University of Jyväskylä, Finland; 3 Department of Physics, Lund University, Lund, Sweden; 4 STFC Daresbury Laboratory, Daresbury, Warrington, WA4 4AD UK; 5 Present Address: Center for Nuclear Study, The University of Tokyo, Hirosawa 2-1, Wako, Saitama 351-0198 Japan

## Abstract

Nuclear physics experiments often involve complex geometrical configurations and intricate de-excitation schemes. Combined $\gamma$-ray and conversion-electron spectroscopy experiments are prime examples. The design of the required instrumentation and the interpretation of the resulting data can greatly benefit from detailed simulations. Here, we report on extensions to the NPTool framework that enable an accurate representation of the experimental conditions associated with the sage and spede spectrometers. In addition, we introduce a program package for implementing complicated de-excitation patterns to complement the NPTool framework.

## Introduction

In recent decades, nuclear spectroscopic techniques have benefited from the substantial advancement of detector technology, evolving from single detectors connected to analogue single-channel analysers to integrated detector arrays utilising digital electronics. This expansion has allowed for investigations of rarer signals, but has also increased data complexities, necessitating advancements in tools for interpretation. Geant4 [1], originally developed to simulate high-energy physics experiments, has emerged as a key asset in several physics disciplines. In nuclear physics, implementations of Geant4 have been developed to better exploit this powerful toolkit. One such framework, NPTool [2], was created to allow simulations to be performed without requiring a re-implementation of Geant4 each time. NPTool uses a modular approach, with a growing library of detectors being created by the community and incorporated into the toolkit. In this way, with little prior knowledge, a user can construct an experimental setup from existing detectors and use the NPTool framework to generate a reaction, simulate interactions with active (e.g. germanium crystal) and inactive (e.g. detector capsule) material of detectors, and produce data in the ROOT framework [3] that can be easily analysed or compared with experimental data.

**Fig. 1:**
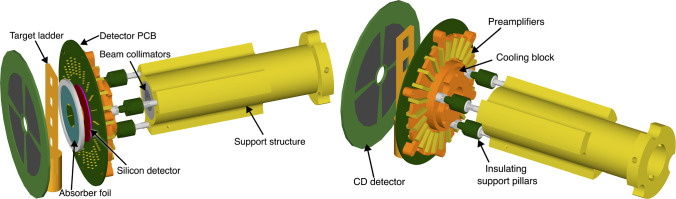
Front and rear view of the simulated geometry of spede inside the target chamber, including, the Miniball CD detector, target ladder, absorber foil, silicon detector mounted on the PCB, the preamplifiers, the cooling block, electrically and thermally insulating support pillars, the primary beam collimators, and the support structure

In this paper, we present a number of additions to the NPTool framework motivated by the need to simulate data obtained with in-beam $\gamma$-ray and electron spectrometers, such as the sage [4, 5] and spede detector systems [6]. In particular, we have introduced the conversion of RadWare-format [7] level schemes into Geant4-format PhotonEvaporation files, to facilitate their accurate and quick simulation. Electromagnetic transitions that include an electric monopole (*E*0) component required special consideration, since they have not been natively implemented in RadWare or Geant4. Therefore, we present a method for the treatment of such transitions.

## Construction of the simulation

### Introduction of geometries and electromagnetic fields in the simulation

The simulation package was developed within the NPTool framework, which provides a major advantage: a wide range of pre-implemented detectors contributed by users, including the Miniball cluster and CD detectors [8], both essential for simulating in-beam experiments employing the spede spectrometer. NPTool’s modular design allows detectors to be combined within a setup using a simple detector description file. This enables users to position detectors efficiently and rapidly, without the need to recompile the main code. The additions made to the NPTool framework in this work are available to all NPTool users and include the Phase I- and Clover-type germanium detectors of the jurogam array [9], the sage and spede spectrometers, as well as enhancements to the Miniball array geometry as described later in the text. In addition to its simulation capabilities, NPTool also provides an integrated analysis framework that supports the simultaneous processing of both experimental and simulated data for direct comparison.

The modular structure of NPTool, combined with the flexibility of Geant4, enables the development of a user-friendly simulation package featuring a simple and interactive interface. This interface eliminates the need for extensive knowledge of Geant4 or C++, making it accessible to a broad range of users.

To facilitate direct control over key experimental parameters, a set of configurable variables has been implemented. These are described in detail throughout the text as needed:

- position and thickness of the sage and spede silicon detectors,
- position of individual germanium detectors, defined relative to the target position and beam axis,
- inclusion and properties (inner radius, foil thickness) of the sage carbon-foil unit,
- position and properties (material, thickness) of the spede absorber foil,
- option to activate the jurogam BGO shields to veto Compton-scattered $\gamma$ rays,
- target specifications (material, thickness, and backing),
- position of the Miniball CD-type particle detector, and
- electromagnetic field table file and associated scaling factor.

**Fig. 2:**
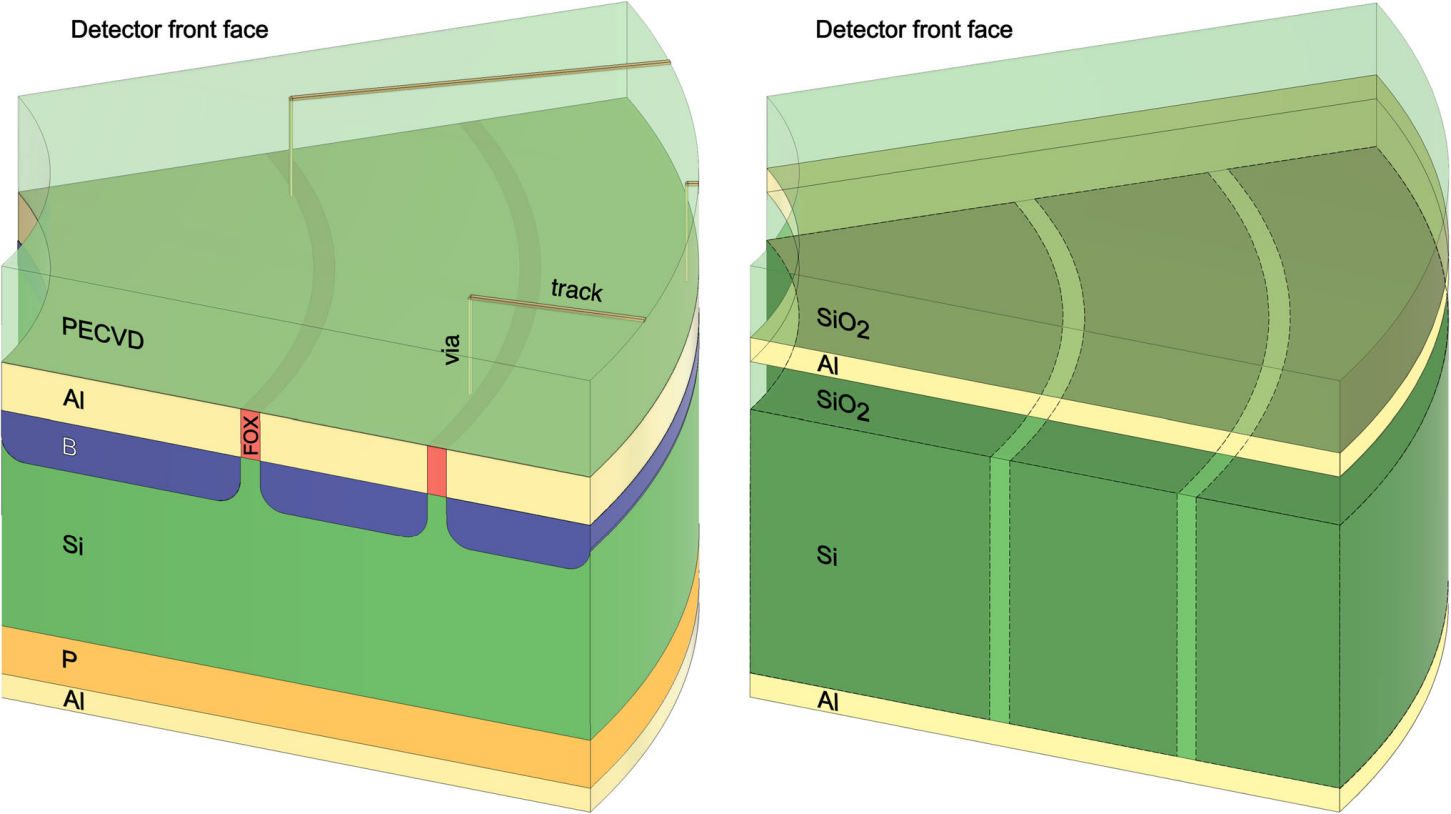
**Left:** Schematic representation of a portion of the spede segmented silicon detector using the double-metal process to route signals to the detector periphery. The rear of the silicon bulk is doped with phosphorus and the front with boron. Aluminium is deposited on both sides, following the shape of the active segments on the front, while on the rear a uniform layer is placed. The aluminium segments are separated by SiO$_2$ Field Oxide (FOX). SiO$_2$ is also deposited on the front face (PECVD) (transparent in the figure), to electrically separate the aluminium signal tracks from the active segments. Each track is connected to a single segment through a via. The guard rings are omitted from the drawing for simplicity. **Right:** The approximation used in geant4 for the same portion of the spede silicon detector. Successive uniform layers of SiO$_2$, aluminium and SiO$_2$ (transparent in the figure) are placed on top of the silicon bulk, while a uniform layer of aluminium is placed on the back. A scorer was used to emulate the pixel segmentation (see text for details). The active pixel boundaries are represented with dashed lines and a darker shade. The doping of the silicon bulk and the guard rings were not included in the simulation. In both figures, the layers are not to scale

For each simulated experimental setup, all components relevant to the tracking and detection of both $\gamma$ rays and electrons, or those that could potentially interfere with their paths, were included in the simulation. To ensure both simplicity and accuracy, most non-detector geometries were imported directly from technical drawings using the CADMesh package [10]. As an example, Fig. 1 shows the simulated geometry of the target region of the spede spectrometer. The level of detail included in the simulation is evident in the figure, which features individual preamplifiers, the copper cooling block, steel and plastic mounting pillars, the brass support structure, a two-part (lead and tungsten) beam collimator, and a target ladder constructed from PEEK plastic. Special attention was given to the design and placement of these components, as they can obstruct the path of $\gamma$ rays to the Miniball cluster detectors, reducing the spectrometer’s $\gamma$-ray detection efficiency.

The silicon detectors of the sage and spede spectrometers are segmented into 90 and 24 segments, respectively, and employ a double-metal design to route signals from the active segments to the detector periphery [11]. This segmentation, along with the double-metal structure, introduces narrow inactive regions between the segments as well as thin inactive layers on the front face of the detector. For completeness, the complex structure of these detectors is outlined in the following, along with the approach taken to model them in the simulation.

Detector fabrication involves several key steps, based primarily on photolithography, etching, ion implantation, and deposition techniques [11]. The various layers are successively built on a polished silicon wafer using patterned masks. On the front face, the active segments are doped with boron (acceptor) and then covered with a thin aluminium layer (first metal, 0.6±0.1 $\upmu$m) shaped to match each segment. This metal layer is embedded in a field oxide (FOX, SiO$_2$), typically 0.9±0.1 $\upmu$m thick, with a width of 7 $\upmu$m for sage and 50 $\upmu$m for spede. The detector surface is then coated with a uniform 3.5 $\upmu$m layer of SiO$_2$ using Plasma-Enhanced Chemical Vapour Deposition (PECVD). Thin aluminium tracks (second metal, 1.5  $\upmu$m thick) are deposited on top of this insulating layer and connected to the first metal via etched contact holes (vias). These tracks route signals to bonding pads located outside the active area.

The rear surface of the detectors is unsegmented and uniformly doped with phosphorus (donor). It is coated with a 0.3 $\upmu$m-thick aluminium layer. Additionally, the detector edges are equipped with multiple guard rings (on the outer edge for sage, and on both the inner and outer edges for spede) to reduce leakage currents and improve performance. A schematic of the spede silicon detector structure is shown in Fig. 2, left.

Although it is not necessary to replicate the full complexity of this structure in the simulation, special care was taken to approximate its various layers. A uniform silicon disk was used to represent the silicon bulk, overlaid by three successive uniform layers, 0.9 $\upmu$m of SiO$_2$, followed by 0.6 $\upmu$m of aluminium and then 3.5 $\upmu$m SiO$_2$. Additionally, a uniform 0.3 $\upmu$m-thick aluminium layer was added on the rear side.

The segmentation of the silicon detector was emulated using a scorer in geant4. This allows the construction of complex sensitive geometries, including inter- and intra-ring dead layers. This approach also simplifies the recording of particle interactions and precise energy depositions within the material. To define the segmentation of the detector in the scorer, polar coordinates were used. The inner and outer radii and the start and stop angles for each detector pixel were recorded in a geometry file. For each simulation step, if energy is deposited in the silicon bulk, then this is recorded if the deposition point falls within pixel boundaries, otherwise the energy is not recorded and it is considered that the deposition point is in an inactive segment. After running a full event in geant4, the energy deposited in each pixel is summed and reported. These approximations are visualised in Fig. 2, right, and were validated by successfully reproducing the alpha spectra measured with the spede detector at the ISOLDE Decay Station (IDS).

To mimic the various sources of electronic noise encountered under typical experimental conditions, such as bulk and surface leakage currents, as well as noise from signal-chain components, including preamplification and amplification stages, a user-defined randomisation of the detected energy was applied to better match the measured detector resolution.

The jurogam and Miniball germanium detector arrays were also modelled in the simulation, with each individual detector module constructed and oriented to reproduce the configurations used in real experiments. In the jurogam frame, each tapered (Phase 1- or GASP-type) and Clover detector is mounted in a fixed position and orientation at a defined target-to-detector distance, as described in Ref. [9].

In contrast, the Miniball spectrometer is a highly flexible array that allows each of the eight germanium cluster detectors to be positioned at variable angles and distances from the target within certain limits. In addition, the cluster detectors are electrically segmented. All of these features have been implemented in the simulation.

jurogam is primarily used in fusion-evaporation reaction experiments, where its germanium detectors are exposed to neutron fluxes that can displace atoms in the crystal lattice. These defects lead to incomplete charge collection, reducing spectral quality, and are typically mitigated through detector annealing. However, repeated annealing cycles can cause lithium donor atoms to diffuse deep into the crystal, further lowering the performance. Restoring the crystal requires removing a thin outer layer by lapping, followed by fresh lithium diffusion and re-establishment of electrical contacts. This process reduces the volume of germanium crystals and consequently the detector’s geometrical efficiency. Several jurogam detectors have undergone this treatment and so their reduced volume has to be accounted for in the simulations. However, since the exact post-treatment dimensions are unknown, an average correction has been applied to all detector crystals and validated using full-array efficiency measurements with calibration sources [5].

Both sage and spede suppress $\delta$-electron fluxes generated by beam–target interactions using electric fields, while sage additionally employs a solenoidal magnetic field for electron transport. These fields were modelled using the opera 3d simulation package [12], mapped on a three-dimensional grid and imported into geant4. This approach was preferred over direct analytical field computation in geant4, as it is less computationally intensive and significantly reduces simulation runtime. The fields can be scaled by the user according to experimental conditions, including the voltage used on the spede target ladder, the sage high-voltage barrier and the current in the sage solenoid coils. Any changes in field-sensitive components, such as the positioning or thickness of magnetic materials, require the generation and import of new field maps.

### Kinematic correction of internal conversion electrons and $\gamma$ rays

The internal conversion electrons (ICE) and $\gamma$ rays detected in the experimental setups discussed in this paper are typically emitted by nuclei in motion. Consequently, their observed energies must be corrected for reaction kinematics. For $\gamma$ rays, this procedure is commonly referred to as Doppler correction.

geant4 provides several physics lists to model different physical processes, including the kinematic correction of particles emitted in flight. To perform these corrections accurately, NPTool allows the user to specify the relevant reaction parameters. For two-body reactions, such as the Coulomb excitation reactions typical of spede, these include:

- beam particle properties (type, energy, and energy spread),
- beam spot size and position,
- target material, thickness, and backing,
- differential reaction cross-section as a function of centre-of-mass angle, and
- masses, proton numbers, and excitation energies of the light and heavy reaction products.

The fusion-evaporation reactions typical for sage experiments, can also be approximated as two-body reactions in NPTool, provided that mass and charge conservation are respected. If the reaction produces more than two products (e.g., a heavy nucleus plus light particles such as protons, neutrons, or $\alpha$ particles), the lighter products can be treated as a single effective nucleus in the simulation. Since in the simulation only the de-excitation of the heavy nucleus will be considered, for which the differential cross-section can be defined by the user, this approximation yields realistic results. Future NPTool releases may include explicit treatment of fusion-evaporation reactions.

As an example, in a typical simulation of a sage experiment employing a fusion-evaporation reaction, the user can define the beam–target interaction region as a disc centred at the production target position, with a radius equal to the beam radius. The heavy reaction products can be assigned the same initial direction as the beam, and the emitted $\gamma$ rays and ICE will be generated isotropically in the rest frame of the heavy reaction product. A feature for simulating angular emission patterns based on $\gamma$-ray multipolarity is currently being developed by the geant4 collaboration and could be integrated into future simulations.

A similar approach applies to Coulomb excitation reactions. In this case, the light and heavy reaction products correspond directly to the incoming beam and target particles. For these simulations, it is preferable to generate both reaction products, as they are often both measured experimentally, and excitations can occur in either nucleus depending on the physical conditions.

## Simulation of de-excitation paths

### Generic case

Radioactive decays of nuclei with proton numbers $Z \le 100$, and with level schemes published in the ENSDF database [13], are included in the standard geant4 data files. This restriction limits the applicability of the simulation package, since the published level schemes for exotic nuclei are often incomplete, and sage and jurogam experiments routinely involve nuclei with proton numbers $Z > 100$. A development is ongoing by the geant4 collaboration to include nuclei with proton numbers $Z > 100$ [14], however, this functionality is not yet part of the standard geant4 distribution.

To address these limitations and enhance the versatility and usability of the simulations, we have developed an extension to NPTool, which enables the generation of geant4-compatible decay data files from RadWare-format level schemes [7]. Additional Python tools were implemented to:

- Extract measured or estimated transition intensities from RadWare level schemes,
- automatically calculate internal conversion coefficients (ICCs) using the BrIcc conversion coefficient calculator [15], and
- treat transitions with an *E*0 component explicitly.

Explicit treatment of *E*0 transitions was necessary due to limitations in both RadWare and geant4. For *E*0 transitions, single $\gamma$-ray emission is forbidden, and in most situations the emission of two simultaneous $\gamma$ rays is a negligible higher-order process. Such transitions proceed only via internal conversion or, if energetically allowed, electron–positron pair emission. However, RadWare considers only $\gamma$-ray transitions and geant4 assumes that all photon emissions have a finite ICC, and thus cannot natively simulate pure *E*0 transitions. These limitations are particularly important for sage and spede experiments, which often focus on studies involving *E*0 transitions. The developments reported here represent an expansion and refinement of the approach first described in [5].

The procedure for creating realistic de-excitation patterns for the nuclei of interest is presented below, while the explicit treatment of transitions with an *E*0 component is discussed in Sect. 3.2. All scripts were developed as part of this work, unless otherwise noted, and can be found together with detailed instructions, in the repository available via Ref. [16].

The procedure for generating simulation input files for a single nucleus consists of the following steps:

1. **Create the level scheme** of interest in RadWare and save it as a Graphical Level Scheme (GLS), e.g., AZ.gls, where *A* and *Z* denote the mass and proton numbers of the nucleus, respectively.
2. **Convert the GLS file** to a human-readable Ascii-GLS (AGS) file using the RadWare program gls_conv, producing, for example, AZ.ags.
3. **Extract and process level information** using the readfile.py script. This script:
  Reads level and transition data from the AGS file,
  calls the BrIcc calculator [15] to compute ICCs, and
  combines this information into a file named user_zZ.aA following the geant4 naming convention, where *Z* and *A* are replaced by user-provided values.
  The resulting file contains one data block per level, in standard geant4 format, with:
  A line describing the level by:
    a unique identifier,^1^
    its excitation energy,^2^
    half-life (set to -1 for the ground state and 0 for all other levels),^3^
    spin, and
    the number of distinct $\gamma$ rays de-exciting the level.
  A line for each $\gamma$ ray transition de-exciting the level, specified by:
    the level it feeds (identified by the unique identifier),
    the transition energy,
    the transition intensity,
    the transition multipolarity,
    the multipole mixing ratio, and
    the total and partial ICCs of the transition.
    The multipolarity notation and the sequence of partial ICCs are described in readfile.py.
  As an illustration, a single-level data block from user_zZ.aA might appear as follows. For simplicity, information of only the first two levels in the example are included and also higher order partial ICCs have been omitted. The complete file can be found via the link in Ref. [16]. 
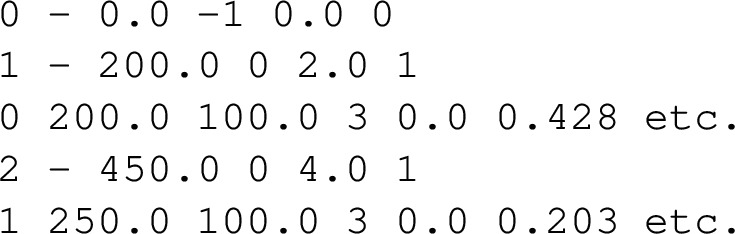
4. **Calculate feeding of initial levels** using
  InitialPopCalculator.py. This script evaluates the balance of incoming and outgoing transition intensities for each level, using the values in user_zZ.aA. The resulting initial feeding values are normalised so their sum equals one and are written to the file initial_population_zZ.aA, representing the fraction of nuclei produced in each level following the nuclear reaction.
  The initial_population_zZ.aA file taken from the same example is shown below, where the first column indicates the level energy and the second the initial feeding. 
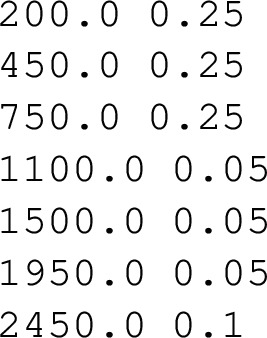
5. **Provide the generated files to the simulation.** The two output files (user_zZ.aA and initial_population_zZ.aA) fully describe the de-excitation scheme of a particular isotope and are supplied to the simulation via user-defined variables in the reaction file, which specifies the beam–target interactions (see Sect. 2.2).

For simulations involving multiple nuclei, these steps are repeated independently to produce input files for each nucleus. Each nucleus can be simulated separately, and then the resulting outputs can be combined and weighted according to the calculated or measured production cross sections.

### Treatment of transitions with an *E*0 component

Because RadWare does not inherently handle transitions with *E*0 components, these must be treated separately to correctly account for initial level feeding in the simulation. For this work, transitions with *E*0 components were classified into two categories:

- Pure *E*0 transitions, and
- mixed $E0+M1$ or $E0+E2$ transitions.

Transitions of $E0+M1+E2$ mixed character were not considered in this study. The procedure described below was validated by successfully reproducing the experimental data obtained from a study of $^{188}$Pb with the sage spectrometer [17].

#### Pure *E*0 transitions

To simulate a pure *E*0 transition (that is, a transition between two $0^+$ states), a virtual $\gamma$ ray is introduced into the RadWare level scheme connecting the two levels. The virtual $\gamma$ ray is assigned with:

- The minimum allowed intensity value, and
- a large total ICC to ensure the *E*0 transition intensity balances correctly with the relative intensities of other transitions de-exciting the $0^+$ state that is higher in energy.

After saving the modified level scheme and converting it to AGS format (steps 1 and 2 in Sect. 3.1), the multipolarity of the virtual $\gamma$ ray is manually changed to *E*0 in the AGS file. This enables BrIcc to compute the correct electronic factors for the *E*0 transition.

Finally, by following steps 3 and 4 from Sect. 3.1, the simulation input files are generated. Accordingly, the resulting initial_population_zZ.aA. file incorporates pure *E*0 transitions.

#### Mixed *E*0 transitions

For mixed *E*0 transitions, the partial ICCs must be adjusted to ensure that the simulated $\gamma$-ray and ICE intensities correctly reproduce those of the mixed transition. As an example of how this is applied in the simulation, the case of a mixed $E0+E2$ transition will be considered.

The total transition intensity consists of the $\gamma$-ray intensity associated with the *E*2 component, and the ICE intensities associated with both the *E*0 and *E*2 components.

The ICE intensity is further distributed among all energetically allowed atomic shells, with relative contributions determined by the ICCs ($\alpha _S$) for the *E*2 component and the electronic factors ($\varOmega _S$) for the *E*0 component, where S represents the atomic shell.

The challenge is to determine combined ICCs that reflect the $E0+E2$ character of the transition. geant4 requires two quantities for each mixed transition, the total ICC, $\alpha _T(E0+E2)$, and the fractional ICC for each atomic shell defined as:

1 $$\begin{aligned} \frac{\alpha _S(E0+E2)}{\alpha _T(E0+E2)}. \end{aligned}$$

Experimentally, the observables typically considered as the starting point, are the measured $\gamma$-ray intensity, $I_\gamma$ (in the units used in the RadWare level scheme) and the measured K-shell ICC, $\alpha _K^{mes}$. The $\gamma$-ray intensity is added to the level scheme in RadWare, and the de-excitation information is extracted following steps 1.-3. of Sect. 3.1. At this stage, the transition is still denoted as a pure *E*2 transition in the level scheme, so the ICCs computed by BrIcc are incorrect. To obtain the correct combined ICCs for the mixed transition, the *E*2 ICCs, $\alpha _S(E2)$, the *E*0 electronic factors, $\varOmega _S(E0)$, and the relative intensities of the *E*0 and *E*2 components are used. This procedure has been automated in the script ME0C.C, which only requires $I_\gamma$ and $\alpha _K^{mes}$ as input to produce the correct ICCs for the simulation.

The script first determines the intensity of the *E*2 component, *I*(*E*2), using $I_\gamma$ and $\alpha _S(E2)$. The excess electron intensity, responsible for the larger $\alpha _K^{mes}$, is attributed to the *E*0 component. The corresponding *E*0 intensity, *I*(*E*0), is then calculated using an effective ICC, $\alpha '(E0)$, according to the equation:

2 $$\begin{aligned} I(E0) = I_{\gamma }\,\alpha '(E0) = I_{\gamma }\,\frac{\alpha _K^{mes}-\alpha _K(E2)}{\varOmega _K(E0)/\varOmega _T(E0)}. \end{aligned}$$

The fractional ICC for each atomic shell is evaluated by dividing the electron intensity from that shell by the total electron intensity via the expression:

3 $$\begin{aligned} \frac{\alpha _S(E0+E2)}{\alpha _T(E0+E2)}\,I_{e^-} = I_{e^-S} = I_\gamma \,\alpha _S(E2)\,+\,I(E0)\,\frac{\varOmega _S(E0)}{\varOmega _T(E0)}. \end{aligned}$$

The $\alpha _S(E0+E2)/\alpha _T(E0+E2)$ ratios calculated through this process should replace the ICC fractions which were calculated using BrIcc in the user_zZ.aA file. Following this correction, step 4. of Sect. 3.1 will produce the correct initial feeding of the levels.

## Example of a simulated band

To illustrate the capabilities of the NPTool extensions developed in this work, we present a case study comparing them to data obtained in an in-beam spectroscopy experiment investigating the neutron-deficient nucleus $^{188}$Pb. In [17], a total of 12 nuclei were simulated independently and subsequently combined to reproduce the spectra observed in an experiment employing the $^{160}$Dy($^{32}$S,4n)$^{188}$Pb reaction and the sage spectrometer.

**Fig. 3:**
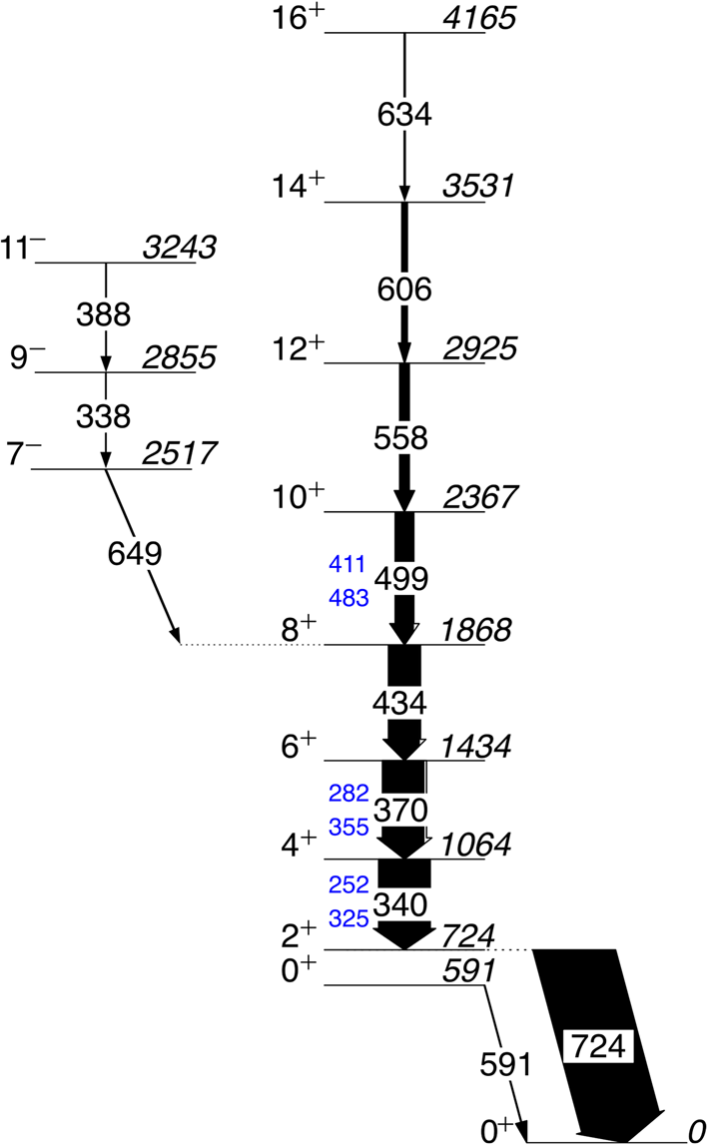
Partial level scheme of $^{188}$Pb showing the most prominent transitions that were observed in coincidence with the $8^+_1 \rightarrow 6^+_1$ 434 keV yrast-band transition. K (top) and L (bottom) internal conversion electron energies are marked in blue

**Fig. 4:**
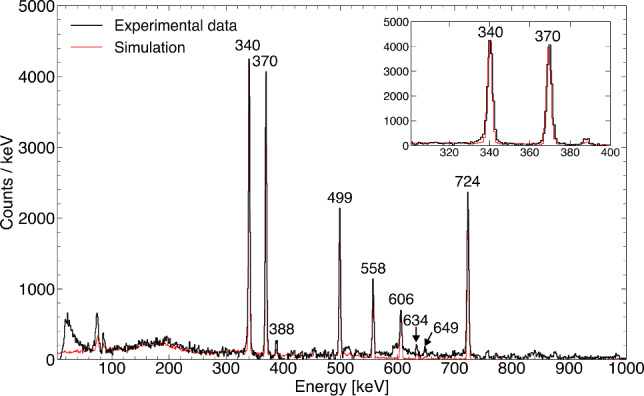
Simulated (red) and measured (black) $\gamma$-ray energy spectra gated on the 434 keV $\gamma$ rays. Prominent peaks associated with the yrast-band transitions in $^{188}$Pb are labelled according to the transition energy. The inset shows an expansion of the area of the two most prominent peaks

**Fig. 5:**
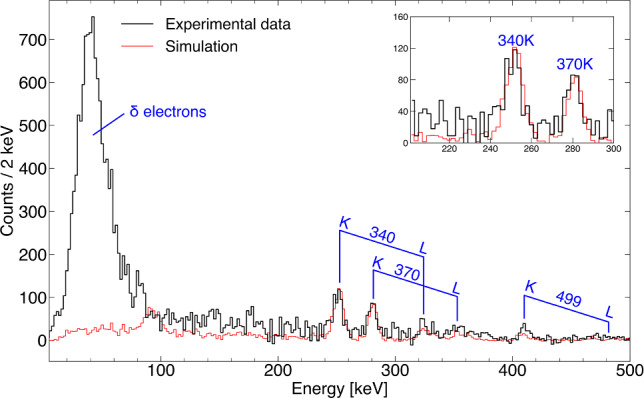
Simulated (red) and measured (black) electron energy spectra gated on the 434 keV $\gamma$ rays. Prominent peaks associated with the yrast-band transitions in $^{188}$Pb are labelled by the corresponding transition energy and the electron’s shell of origin. The inset shows an expansion of the area of the two most prominent peaks

The combined simulation is used here to reproduce transitions in the yrast band of $^{188}$Pb, as shown in Fig. 3. To isolate this band from the dominant background, a gate was applied on the 434 keV $8^+_1 \rightarrow 6^+_1$ transition in both the experimental and simulated $\gamma$–$\gamma$ and $\gamma$–electron energy matrices. The resulting background-subtracted $\gamma$-ray and electron spectra are presented in Figs. 4 and 5, respectively, and demonstrate good agreement between simulation and experiment for this complex case.

In the $\gamma$-ray energy spectrum, the two most prominent peaks are accurately reproduced, whereas minor discrepancies remain at higher energies, primarily due to the intricate feeding pattern in this nucleus. The electron spectrum is reproduced with high accuracy, with the main deviation occurring below 100 keV, where the measured spectrum is dominated by $\delta$-electrons, a process not included in the simulation.

Other examples that demonstrate the quality of the simulation package, including the de-excitation of the 591 keV $0^+_2$ state, can be found in Ref. [17]

## Summary

A simulation package has been developed within the NPTool framework to model experiments employing the sage and spede spectrometers in conjunction with the jurogam and Miniball germanium detector arrays, respectively. Particular attention was paid to accurately reproduce the effective geometries of the active areas of the silicon and germanium detectors, as well as to modeling reaction kinematics. In addition, a suite of Python scripts has been created to generate decay data files compatible with geant4 for cases not natively supported by the package, specifically, nuclei with $Z > 100$ or de-excitation patterns containing *E*0 components. The developments have been validated using the yrast band in $^{188}$Pb.

## Data Availability

The $^{188}$Pb dataset analysed during the current study is available in the Etsin repository, 10.23729/a1927d10-ca20-4aac-81c8-b5a9ae0eff4d.

## Footnotes

1 This corresponds to the position of the level within the level scheme, i.e. the ground state is assigned identifier 0, the next level in energy is assigned identifier 1 and so on.

2 Relative to the bandhead energy in the case of missing transitions to the ground state.

3 This feature is not currently implemented in the simulation. The half-life descriptor in the file has been included for future use.

## Code Availability Statement

The GEANT4 code generated during the current study is available in the Software Heritage archive, https://archive.softwareheritage.org/swh:1:dir:c9db6dc09d7a9e90646cb88feadc6ccd2366158b. The scripts generated during the current study are available in the Software Heritage archive, https://archive.softwareheritage.org/swh:1:dir:96e6da81be6b667b7a0db1b4167f83448d5e5c97. The CADMesh package used during the current study is available in the github repository, https://github.com/christopherpoole/CADMesh.git.
